# The Combination Therapy with Zoledronic Acid and Propranolol Improves the Trabecular Microarchitecture and Mechanical Property in an Rat Model of Postmenopausal Osteoporosis

**DOI:** 10.1155/2014/586431

**Published:** 2014-03-30

**Authors:** Deepak Kumar Khajuria, Rema Razdan, D. Roy Mahapatra

**Affiliations:** ^1^Department of Pharmacology, Al-Ameen College of Pharmacy, Bangalore 560027, India; ^2^Department of Aerospace Engineering, Indian Institute of Science, Bangalore 560012, India

## Abstract

We conducted the present study to investigate the therapeutic effects of propranolol (PRO), alone and in combination with the antiresorptive agent ZOL, in a rat model of postmenopausal osteoporosis. Female Wistar rats were OVX or sham-operated at 3 months of age. Twelve weeks after surgery, rats were randomized into six groups: (1) sham + vehicle, (2) OVX + vehicle, (3) OVX + ZOL (100 **μ**g/kg, i.v. single dose), (4) OVX + ZOL (50 **μ**g/kg, i.v. single dose), (5) OVX + PRO (0.1 mg/kg, s.c. 5 days per week), and (6) OVX + ZOL (50 **μ**g/kg, i.v. single dose) + PRO (0.1 mg/kg, s.c. 5 days per week) for 12 weeks. At the end of treatment study, various bone parameters were evaluated. With respect to improvement in the mechanical strength of the lumbar spine and the femoral mid-shaft, the combination treatment of ZOL and PRO was more effective than each drug administered as a monotherapy. Moreover, combination therapy using ZOL and PRO preserved the trabecular microarchitecture better than single-drug therapy using ZOL or PRO in OVX rats. These data suggest that combination therapy with ZOL plus PRO represents a potentially useful therapeutic option for patients with osteoporosis.

## 1. Introduction

Osteoporosis is a degenerative disease characterized by low bone mass and microarchitectural deterioration of bone tissue, leading to enhanced bone fragility and increased fracture risk. Osteoporosis is considered an important public health issue increasing rapidly in the elderly population [1, 2]. The fractures caused by osteoporosis have clinical and public health impacts, as they are often associated with increased morbidity, mortality, and enormous healthcare expenditure [1]. For those already affected by osteoporosis, timely diagnosis of bone loss, assessment of fracture risk, and selection of optimal treatment at appropriate stages of the disease are very important for effective management of osteoporosis.

Zoledronic acid (ZOL) is a third generation nitrogen-containing bisphosphonate that has been shown to significantly reduce the risk of fractures in patients who receive the once-yearly dosing regimen for the treatment of postmenopausal osteoporosis [3]. ZOL interferes with osteoclastic activity by inhibiting osteoclast formation and osteoclast bone resorptive activities and by inducing osteoclast apoptotic cell death. On osteoclast stimulation of bone resorption, the bisphosphonate is released and internalized by the osteoclasts, interfering with osteoclast formation, function, and survival [4–6]. Moreover, the results obtained from several histologic and microcomputed tomography studies have shown that ZOL can be highly effective in prevention of bone loss in ovariectomized (OVX) rats [7–10]. The use of ZOL therapy has been associated with adverse events, such as osteonecrosis of jaw, renal dysfunction, hypocalcemia, and atrial fibrillation [1, 7–11].

**β**-Blockers are now considered to be the potential candidates of therapeutic drugs under investigation for fracture healing and more specifically for osteoporosis therapy [8–10]. Many genetic and pharmacological studies have shown that activation of **β**-adrenergic receptors in osteoblasts and stromal cells leads to inhibition of bone formation and intensification of bone resorption [12–15]. In an animal study, a lower dose of propranolol (PRO), a nonselective **β**-blocker, has been shown to increase bone mass in different experimental models of bone disorders [8–10, 16–18]. Results of some prior epidemiological studies confirm the hypothesis that **β**-blockers use is associated with a decrease in fracture risk [19–21].

Combination drug therapy seems to be promising because, in some cases, it can increase the effectiveness of treatment and is now the subject of extensive investigation [22, 23]. Rodrigues et al. demonstrated that low doses of PRO suppress bone resorption by inhibiting receptor activator of nuclear factor kappa-B ligand (RANKL) mediated osteoclastogenesis as well as inflammatory markers without affecting haemodynamic parameters [24]. This result is supported by a previous finding, which showed that propranolol stimulates osteoprotegerin (OPG) on its own in osteoblast cells [25]. PRO, which could directly prevent bone loss and biomechanical alteration by increasing bone formation and decreasing bone resorption, may be the next osteogenic agent for osteoporosis treatment [8, 16, 24].

To the best of our knowledge, no study has assessed whether the combination of ZOL and PRO enhances bone properties in rat model of experimental osteoporosis. Owing to the different mechanisms of action of ZOL and PRO, our hypothesis was that the combination of ZOL and PRO would facilitate greater improvements in bone properties than either intervention alone.

## 2. Materials and Methods

### 2.1. Drugs, Chemicals, and Other Materials

ZOL was obtained from Naprod Life Sciences, Maharashtra, India. PRO, ketamine, xylazine, and xylene were obtained from Aurobindo Pharma (Hyderabad, India), GlaxoSmithKline Pharmaceuticals (Mumbai, India), Neon Pharma (Mumbai, India), Indian Immunologicals (Hyderabad, India), and S.D. Fine Chemicals (Mumbai, India), respectively. Ethicon chromic sutures-3/0 and Ethicon mersilk sutures-3/0 were obtained from Johnson & Johnson Ltd., Baddi, Himachal Pradesh, India.

### 2.2. Experimental Animals

In-house laboratory bred healthy female Wistar rats of 3 months of age were included for the study. Animals were maintained under controlled temperature at 25°C ± 2°C with 12 h light/dark cycle with food and water provided ad libitum. The experiments were conducted as per the CPCSEA (Committee for the Purpose of Control and Supervision of Experiments on Animals) guidelines after obtaining ethical clearance from the Institutional Animal Ethical Committee.

### 2.3. Preclinical Study Design

**Figure 1:**
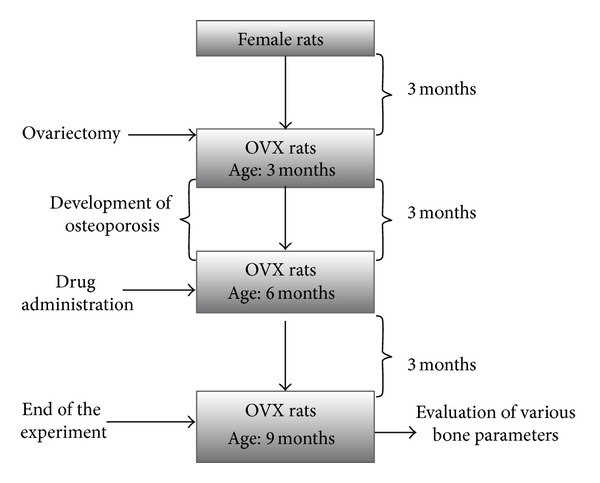
A flow chart showing the preclinical study protocol.

At three months of age, ovariectomy was performed under ketamine and xylazine anaesthesia (80 mg/kg and 10 mg/kg; i.p.) according to the method described by Khajuria et al. [26]. The SHAM-operation rats were subject to SHAM surgery exposure without removing the ovaries. At 3 months after ovariectomy (age: 6 months), animals were divided into 6 groups of 6 animals each. The SHAM group and one OVX group served as negative and positive controls, respectively. A group of OVX rats were left untreated (untreated OVX). Both the untreated OVX and the sham OVX groups were used as controls. Two OVX groups were treated with single intravenous dose of ZOL at 50 *μ*g/kg (ZOL 50 group) and 100 *μ*g/kg (ZOL 100 group), administered into tail vein as a slow intravenous injection over 30 s under light inhalation anesthesia. One OVX group was treated with PRO at a dose of 0.1 mg/kg, injected subcutaneously five days per week, for 12 weeks. Treatment on the remaining OVX group was initiated with a 50 *μ*g/kg ZOL and 0.1 mg/kg PRO combination-treated (ZOL 50 + PRO) group. Subcutaneous injections five days per week in case of OVX groups treated with PRO and ZOL 50 + PRO require some animal handling and create some stress to the animals. Therefore, apart from positive and negative control groups, OVX groups treated with ZOL (100 *μ*g/kg and 50 *μ*g/kg, intravenous single dose) were also subcutaneously administered vehicle (normal saline, 5 days per week) for 12 weeks. The medication dosages used in this experiment were selected from previous studies on rat osteoporosis model. In our previous study, we used 100 *μ*g/kg single intravenous injection of ZOL in OVX rats and showed that this dose of ZOL restores microstructural and biomechanical features of bone. For this reason, 100 *μ*g/kg single intravenous injection of ZOL was used in this experiment [8, 9]. Similarly, we showed that subcutaneous administration of 0.1 mg/kg PRO given 5 days per week for 10 weeks prevents OVX induced bone loss by increasing bone formation and decreasing bone resorption, and thus 0.1 mg/kg given for 5 days per week of PRO was selected in this study [8, 9]. Body weight (expressed in grams) was monitored at the end of the experiments. All groups were euthanized by an overdose of anesthesia. In all rats, femurs and lumbar vertebrae (LV4) were excised and cleared of fat and connective tissues. Femurs and LV4 bones were soaked in saline solution gauze and frozen at −20°C for the microarchitectural and biomechanical tests (Figure 1). The procedure for killing of the animals was in accordance with the CPCSEA (Committee for the Purpose of Control and Supervision of Experiments on Animals) guidelines.

### 2.4. Microcomputed Tomography (Micro-CT) Analysis of Bone

The distal region of the right femora was used for three-dimensional Micro-CT analysis. The Micro-CT and software used for this experiment were from Scano Medical (micro-CT40, Scanco Medical AG, Bassersdorf, Switzerland). Analogous to the Micro-CT measurements, the distal femoral metaphysis was scanned, whereby in this case with a higher isotropic voxel size resolution of 16 *μ*m. In total, 120 high-resolution slices (=2 mm length, same length as chosen for the Micro-CT) with a pixel matrix of 2048 × 2048 were measured using an effective energy of 70 keV and a current intensity of 114 mA. After preprocessing the images with the same Gaussian filter (*∑* = 0.7, support = 1), a threshold was selected at 28% of the maximal grey-scale value, which corresponds to the peak for bone tissue in the histogram of the grey value distribution in the image. Bone volume (BV/TV, fractional bone volume), trabecular number (Tb.N), trabecular thickness (Tb.Th), and trabecular separation (Tb.Sp) were evaluated. In addition to the computation of metric parameters, values of nonmetric parameters were calculated to describe the 3D nature of the trabecular bone sample. The structure model index (SMI) quantifies the plate—rod characteristic of 3D trabecular structure.

### 2.5. Biomechanical Bone Strength Testing

The structural properties of left femurs and LV4 were determined by three-point bending or compression, respectively, using a universal testing machine (BISS Makron, Bangalore, India).

#### 2.5.1. 3-Point Bending Test

Femur strength was assessed by 3-point bending as previously described [8]. Briefly, femurs were removed from the −20°C freezer and rehydrated in a saline solution for 4 h at room temperature. Hydrated weight of the bones was determined using a four-decimal place digital scale. Length of the bones was measured with calipers. Specimens were placed on two supports that were separated by a distance of 12 mm and bent until fracture by lowering the crosshead positioned at the mid-shaft at a constant speed of 0.033 mm/s. From the load-displacement curve, the peak load (N), the ultimate stiffness (N/mm), and the toughness (mJ) were obtained. Ultimate stress (strength) and Young's modulus were derived from load-deformation curves obtained by using equations described by Khajuria et al. [8].

#### 2.5.2. Compression Test

To assess the biomechanical properties of an individual lumbar vertebra, briefly, the LV4 body was separated from the epiphyseal ends, the posterior pedicle, and the spinous process using a low speed diamond band saw. By removing the cranial and caudal ends of the specimen, the planoparallel surfaces were obtained for compression testing. An electronic caliper was used to determine the dimensions of the LV4 body. From the vertebral body, a central cylinder with planoparallel ends and a height of approximately 6 mm was obtained. A vertical compressive force was applied to the specimen in the craniocaudal direction using steel compression plates at a deformation rate of 0.033 mm/sec. The ultimate compressive load (N), the stiffness (N/mm), and the toughness (mJ) were calculated as the mechanical properties from the load-displacement curve. The modulus of elasticity for a material is the slope of its stress-strain plot within the elastic range. The load-deflection curve was converted to stress-strain curve by dividing force by the initial area of the vertebral body and deflection by the original length of the same specimen [9].

### 2.6. Statistical Analysis

All data were expressed as the mean ± standard deviation (SD). For all the data, comparisons between different treatments were analyzed by one-way ANOVA followed by Tukey's multiple comparison tests. In all cases, a probability error of less than 0.05 was selected as the criterion for statistical significance. Graphs were drawn using Graph Pad Prism (version 5.0 for Windows).

## 3. Results

### 3.1. Effects on the Body Weight

The final body weights were significantly greater for animals in the OVX treatment and untreated groups compared with the SHAM animals. There were no statistically significant differences in weights observed between any of the active treatment groups and that of the untreated OVX control group (Table 1).

### 3.2. Trabecular Structure Parameters Using Micro-CT

**Figure 2:**
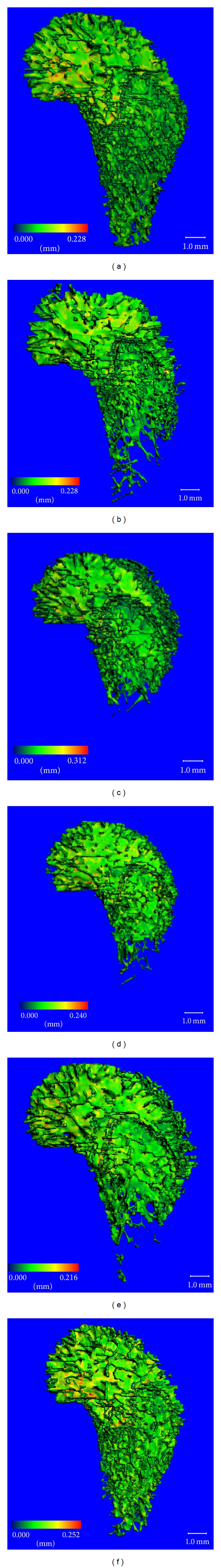
Three-dimensional architecture of trabecular bone within the distal metaphyseal femur region. (a) SHAM group. (b) OVX group. (c) OVX + ZOL 100 group. (d) OVX + ZOL 50 group. (e) OVX + PRO group. (f) OVX + ZOL 50 + PRO group. All groups except SHAM group undergo ovariectomy.

**Figure 3:**
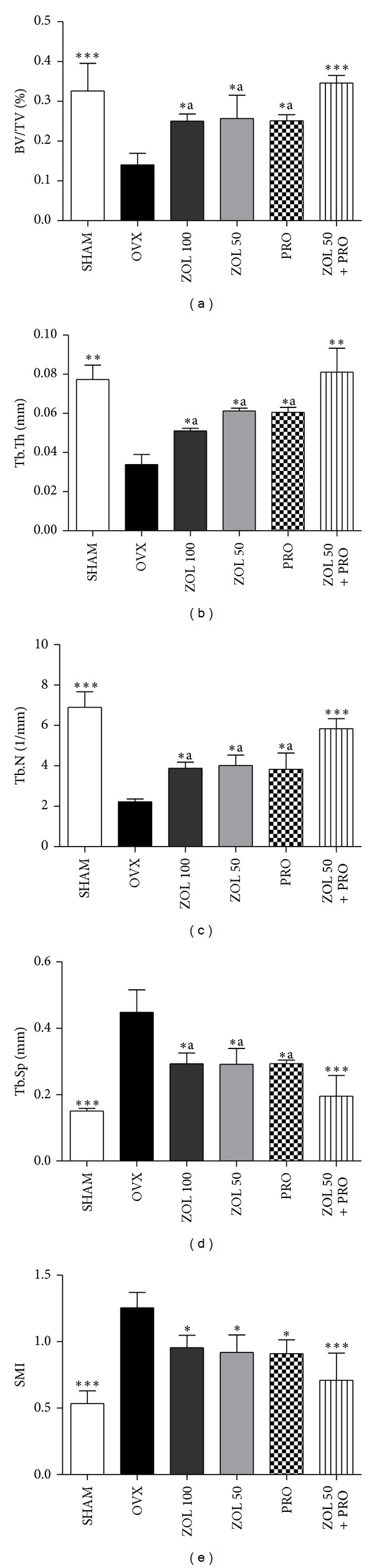
Effects of zoledronic acid and propranolol, alone or in combination, on bone parameters measured by Micro-CT at the distal femur region of ovariectomized rats. Data are shown as the mean ± SD (*n* = 6), evaluated by Tukey's multiple comparison test. *P < 0.05; **P < 0.01; ***P < 0.001, compared to OVX group;  ^a^ *P* < 0.05;  ^b^ *P* < 0.01, compared to ZOL + PRO group. All groups except SHAM group undergo ovariectomy.

3D reconstruction of the trabecular bone structures in the distal femoral metaphysis by Micro-CT is shown in Figure 2. Deterioration of the trabecular architecture due to destruction of trabecular bone of the distal femur was readily observed in OVX group (Figure 2(b)) compared with numerous and well-developed trabeculae in SHAM group (Figure 2(a)). ZOL 100 (Figure 2(c)), ZOL 50 (Figure 2(d)), PRO (Figure 2(e)), and ZOL 50 + PRO (Figure 2(f)) groups prevented bone loss from OVX induced osteopenia. The quantification of trabecular bone changes in the distal femoral metaphysis is shown in Figure 3. As compared to SHAM group, the OVX group showed significant decrease in BV/TV, Tb.Th, and Tb.N. Correspondingly, a significantly higher Tb.Sp and SMI were observed for the OVX group rats compared to that of the SHAM rats, suggesting that ovariectomy caused the trabecular bone to become thin and sparse and disrupted the bone structure. Significant improvements, however, were observed in BV/TV, Tb.Sp, Tb.Th, Tb.N, and SMI values in all single treatments compared with the OVX group. In the ZOL 50 + PRO group, there were significant improvements in these microstructural indices compared with the OVX group. Moreover, the BV/TV, Tb.Th, and Tb.N in the ZOL 50 + PRO group were significantly higher than those in the ZOL 100, ZOL 50, and PRO groups. Correspondingly, a significantly higher Tb.Sp was observed for all single treatment groups compared to that of ZOL 50 + PRO group. Therefore, with respect to improvement of the microstructural parameters of trabecular bone, ZOL and PRO were considered to be independent and additive.

### 3.3. Effects on the Mechanical Properties in the Femoral Mid-Shaft

**Figure 4:**
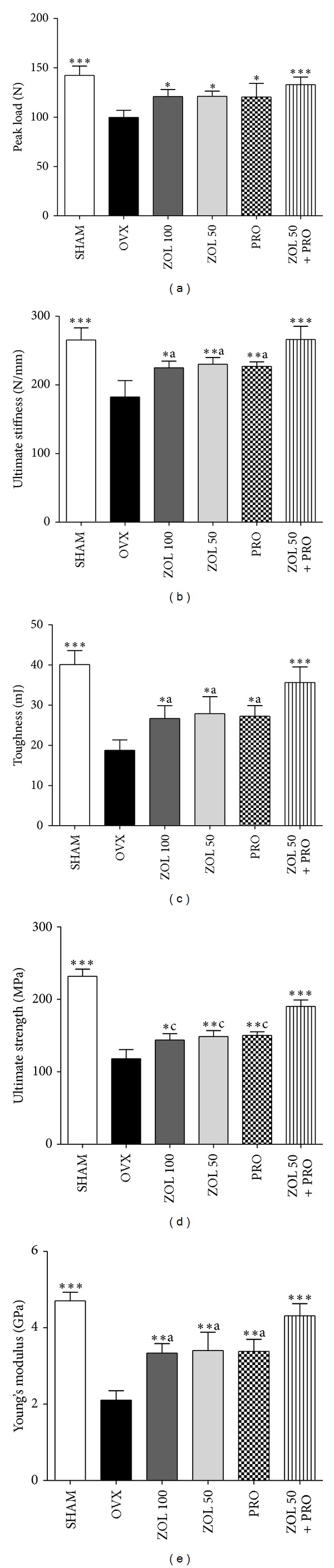
Effects of zoledronic acid, propranolol, or zoledronic acid plus propranolol on the mechanical strength of the femoral diaphysis. The diaphysis was subjected to three-point bending to failure, which provided data on peak load (a), ultimate stiffness (b), toughness (c), ultimate strength (d), and Young's modulus (e). Data are shown as the mean ± SD (*n* = 6), evaluated by Tukey's multiple comparison test. *P < 0.05; **P < 0.01; ***P < 0.001, compared to OVX group;  ^a^ *P* < 0.05;  ^b^ *P* < 0.01;  ^c^ *P* < 0.001 compared to ZOL + PRO group. All groups except SHAM group undergo ovariectomy.

Figures 4(a)–4(e) show the peak load, ultimate stiffness, toughness, ultimate strength, and Young's modulus in the femoral midshaft, respectively. Three-point bending tests of the right femur indicated that ovariectomy caused significant reductions in the peak load, ultimate stiffness, toughness, ultimate strength, and Young's modulus compared with those in SHAM group. In the ZOL 100, ZOL 50, PRO, and ZOL + PRO groups, the peak load of the femur was significantly higher than in the OVX group. Similarly, in the ZOL 100, ZOL 50, PRO, and ZOL + PRO groups, the ultimate stiffness of the femur was significantly higher than in the OVX group. In contrast, the ultimate stiffness in all single treatments was significantly lower than that in the ZOL 50 + PRO group. The toughness of the femur in the ZOL 100, ZOL 50, PRO, and ZOL + PRO groups was significantly higher than in the OVX group. In all single treatments, the toughness was significantly lower than that in the ZOL 50 + PRO groups. Moreover, in ZOL 100, ZOL 50, PRO, and ZOL + PRO groups, the ultimate strength of the femur was significantly higher than in the OVX group. In all single treatments, the ultimate strength was significantly lower than that in the ZOL 50 + PRO group (*P* < 0.001). Furthermore, Young's modulus of the ZOL 100, ZOL 50, PRO, and ZOL + PRO groups was significantly increased when compared with the OVX group. In all single treatments, Young's modulus was significantly lower as compared to ZOL 50 + PRO group.

### 3.4. Effects on the Mechanical Properties in the Fourth Lumbar Vertebra

**Figure 5:**
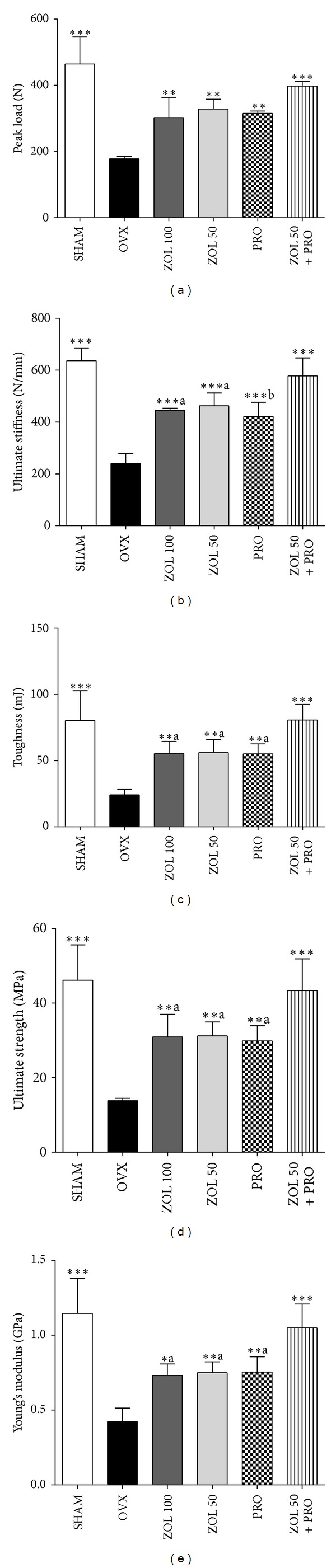
Effects of zoledronic acid, propranolol, or zoledronic acid plus propranolol on the mechanical strength of the fourth lumbar vertebra (LV4). LV4 was compressed to failure to provide data on peak load (a), ultimate stiffness (b), toughness (c), ultimate strength (d), and Young's modulus (e). Data are shown as the mean ± SD (*n* = 6), evaluated by Tukey's multiple comparison test. *P < 0.05; **P < 0.01; ***P < 0.001, compared to OVX group;  ^a^ *P* < 0.05;  ^b^ *P* < 0.0, compared to ZOL + PRO group. All groups except SHAM group undergo ovariectomy.

Figures 5(a)–5(e) show the peak load, ultimate stiffness, toughness, ultimate strength, and Young's modulus in the LV4, respectively. Mechanical compression testing of the LV4 indicated that ovariectomy caused significant reductions in the peak load, ultimate stiffness, toughness, ultimate strength, and Young's modulus compared with those in sham group. In the ZOL 100, ZOL 50, PRO, and ZOL + PRO groups, the peak load of the vertebral body was significantly higher than in the OVX group. Likewise, in all the therapeutic interventions, the ultimate stiffness of the vertebral body was significantly higher than in the OVX group. In contrast, the ultimate stiffness in ZOL 100, ZOL 50, and PRO groups was significantly lower than that in the ZOL 50 + PRO group. Moreover, the toughness and ultimate strength of the vertebral body in the ZOL 100, ZOL 50, PRO, and ZOL + PRO groups were significantly higher than that in the OVX group. In all single treatment groups, the toughness and ultimate strength were significantly lower than that in the ZOL 50 + PRO group. Furthermore, Young's modulus of the ZOL 100, ZOL 50, PRO, and ZOL + PRO groups was significantly increased when compared with the OVX group. In all single treatment groups, Young's modulus was significantly lower than that in the ZOL 50 + PRO group.

## 4. Discussion

The different and complementary modes of action of antiosteoporotic drugs allow a considerable range of combination therapies for treating osteoporosis. In the present preclinical study, we analyzed the skeletal effects of a nonselective **β**-blocker, PRO, in combination with antiresorptive ZOL therapy in OVX rats. We asked whether single intravenous dose of ZOL would (1) inhibit bone loss and microarchitectural changes associated with estrogen deficiency in adult rats and (2) enhance anabolic effects of PRO. Firstly, we clearly demonstrated that combination therapy using ZOL and PRO preserves the trabecular microarchitecture better than single-drug therapy using ZOL or PRO in OVX rats. Secondly and most interestingly, the combination treatment of ZOL and PRO administered improved the mechanical properties of the lumbar spine and femoral midshaft in an OVX rat model of osteoporosis better than single-drug therapy using ZOL or PRO. These findings suggest that the combination treatment of ZOL and PRO had a therapeutic advantage over either the ZOL or PRO monotherapy for treating osteoporosis.

Body weight in the OVX group was greater than in the SHAM group. This indicates that the increase in body weight was caused by ovary extraction. Previous studies from our laboratory and others have shown a similar increase in body weight after OVX, mainly because of the increase in estrogen deficiency [8].

The results of this current study also showed that lower dose of ZOL (50 *μ*g/kg, i.v.) had showed similar promising and beneficial effects in treating osteoporosis when compared with the therapeutic dose of ZOL (100 *μ*g/kg, i.v.) reported by Cheng et al. [27]. As compared to ZOL 100 group, ZOL 50 group has comparable beneficial effects on conserving BV/TV, Tb.Th, Tb.N, and Tb.Sp, which was confirmed by the measurements using Micro-CT in this study. Furthermore, results of the bending and compression test in ZOL 50 group indicate similar ultimate peak load, stiffness, energy, bending stress, and Young's modulus compared to ZOL 100 treated rats. Based on our collective structural and biomechanical analysis, we showed that ZOL 50 may have similar potency in treating osteoporosis in estrogen deficient rats.

Biomechanical testing provides a direct method to study mechanical traits of bones among various experimental animals with different testing protocols [28]. The bone strength is determined by the bone mass and the intrinsic properties of the bone material [8–10]. ZOL plus PRO was statistically superior to all other treatments at increasing stiffness, toughness, ultimate strength, and Young's modulus of the femoral midshaft as well as lumbar vertebrae. This meant that the femora and lumbar vertebrae of rats receiving the combination of ZOL and PRO could resist deformation with the force applied, much better than single-drug therapy using ZOL or PRO.

In the analysis of the architecture of distal femoral trabecular bone using Micro-CT, it was found that combination therapy with ZOL plus PRO was statistically superior to ZOL or PRO monotherapy in suppressing the decrease in BV/TV and the increase in Tb.Sp; both changes are due to ovariectomy. Moreover, combination therapy with ZOL plus PRO was statistically superior to ZOL or PRO monotherapy in increasing Tb.Th and suppressing the decrease in Tb.N, both of which are indices for the trabecular network. These parameters indicate that combination therapy with ZOL plus PRO promotes osteogenesis, thickens trabecular bone, and strengthens trabecular connectivity.

Currently, drugs used in the treatment of osteoporosis are primarily antiresorptive agents, and therefore, PRO, a strong inducer of bone formation [16, 17, 24], is expected to be a useful and effective drug for patients with osteoporosis [19–21]. Studies have demonstrated that low doses of PRO suppress bone resorption by inhibiting RANKL mediated osteoclastogenesis as well as inflammatory markers without affecting haemodynamic parameters [24]. This result is supported by a previous finding, which showed that PRO stimulates OPG on its own in osteoblast cells [25]. Antiosteoporotic activity of PRO (0.1 mg/kg) is associated with no adverse effect on cardiac functions in the rats [16]. This selective action of PRO for bone tissues may be useful in terms of reduced risk of untoward effects observed with ZOL. One of the important factors which may increase renal toxicity of ZOL is higher dose [7, 8, 11, 29]. Serious atrial fibrillation has been reported more frequently in the ZOL-treated patients [1, 8, 30]. Significantly, PRO is among the safest antiarrhythmic drugs currently available which are effective in conversion of atrial fibrillation to normal sinus rhythm [31, 32]. An appealing hypothesis is that PRO when combined with an antiresorptive drug ZOL may result in superior effects on the skeleton and may prevent or reduce the risk of atrial fibrillation. These investigations suggest that the strategy of combined treatment should be a logical and effective therapeutic approach for osteoporosis. In postmenopausal osteoporosis, the tight coupling of bone formation and bone resorption may be compromised by estrogen deficiency which might contribute to cause bone loss. Thus, a combined treatment of promoting this coupling, targeting stimulation of bone formation and inhibition of bone resorption, may have more beneficial effects for improving bone properties in osteoporotic bone.

These results demonstrate that the combined ZOL and PRO therapy can improve the mechanical properties of the spine and femur and preserves the trabecular microarchitecture in an animal model of osteoporosis, suggesting that the combination therapy of ZOL and PRO has a therapeutic advantage over each monotherapy for the treatment of osteoporosis. As such, this combined regimen may be of interest for further evaluation in clinical studies.

## Figures and Tables

**Table 1:** Effects of zoledronic acid, propranolol, or zoledronic acid plus propranolol on body weight.

Group	Body weight (g)
SHAM	304.0 ± 10.65***
OVX	396.6 ± 8.92
ZOL 100	355.0 ± 8.51
ZOL 50	352.0 ± 9.82
PRO	367.0 ± 8.45
ZOL 50 + PRO	359.6 ± 13.14

Data are shown as the mean ± SD (*n* = 6), evaluated by Tukey's multiple comparison test. ***P < 0.001, compared to OVX group. All groups except SHAM group undergo ovariectomy.
